# PKM2 in carcinogenesis and oncotherapy

**DOI:** 10.18632/oncotarget.22529

**Published:** 2017-11-20

**Authors:** Xia He, Suya Du, Tiantian Lei, Xiang Li, Yilong Liu, Hailian Wang, Rongsheng Tong, Yi Wang

**Affiliations:** ^1^ Department of Pharmacy, Sichuan Academy of Medical Science & Sichuan Provincial People's Hospital, Chengdu, Sichuan 610072, China; ^2^ School of Medicine, University of Electronic Science and Technology of China, Chengdu, Sichuan 610054, China; ^3^ Department of Pharmacy, The People's Hospital of Leshan, Leshan, Sichuan 614000, China; ^4^ Institute of Organ Transplantation, Sichuan Academy of Medical Science & Sichuan Provincial People's Hospital, Chengdu, Sichuan 610072, China

**Keywords:** PKM2, Warburg effect, aerobic glycolysis, carcinogenesis, oncotherapy

## Abstract

Tumor cell metabolism is characterized by abundant glucose consumption and aerobic glycolysis. And pyruvate kinase M2 (PKM2) plays a decisive role in glycolysis, significantly contributing to the Warburg effect, tumor growth, angiogenesis, cell division, metastasis and apoptosis. To date, researchers have unraveled the potential of pyruvate kinase M2 as an antitumor target, which suggests a new orientation for oncotherapy. Herein, we focus on the role of pyruvate kinase M2 in tumor cell development and its function as a potential new therapeutic target for tumor treatment. Besides, research actuality on pyruvate kinase M2-dependent glycometabolism and signaling pathway in tumors is also summarized, providing valuable suggestions for further study in this field.

## INTRODUCTION

Pyruvate kinase (PK), including four PK isoforms in mammals: PKL, PKR, PKM1, and PKM2 [1], catalyzes the conversion of phosphoenolpyruvate (PEP) and ADP to pyruvate and ATP in the final step of glycolysis. PKL (predominantly expressed in liver and kidney) and PKR (highly expressed in red blood cells) are both encoded by the same chromosomal gene PKLR [2]. PKM1 is preferentially expressed in adult tissues, whereas PKM2 is abundantly expressed in embryonic tissues and tumors that are proliferating rapidly, as well as in differentiated tissues such as lung, fat, retina and islet [3]. In addition, PKM1 and PKM2 are alternative splicing transcripts of PKM gene (exon 9, PKM1; exon 10, PKM2). During tumorigenesis, the expression of PKM1/L/R are gradually reducing while the expression of PKM2 are greatly increasing, suggesting the unique role of PKM2 in cancer cells [4, 5]. However, some researchers have performed an absolute quantification of PKM1 and PKM2 splice isoforms in plentiful tissue samples including tumor tissues and normal tissues. And they doesn't observe an isoform switch from PKM1 to PKM2 during cancer formation [6]. Similarly, it is indicated in other studies that although PKM2 expression is found to be increased in all examined types of cancer, isoform switch from PKM1 to PKM2 only occurs in glioblastomas. It is also demonstrated that in many types of cancer, elevated PKM2 expression is controlled by the methylation status of PKM gene intron 1 [7]. On the contrary, it is revealed in an experiment that the PKM2/PKM1 ratio was further elevated through the targeting of polypyrimidine tract-binding protein 1 (PTB1) by microRNA-124 (miR-124) and miR-133b, which is regarded as a switch of PKM isoform from PKM2 to PKM1 [8]. The alternative splicing of PKM has been controversial for a long time, but many scientists now tend to approve the increase of PKM2 expression in tumor.

Although PKM1 and PKM2 differ only in 23 amino acids, they play distinct role in the regulation of cellular glycometabolism [9]. PKM1 generally forms a stable tetramer with high pyruvate kinase activity in order to catalyze the conversion of PEP and ADP to pyruvate and ATP [10]. Consequently, the product would be translocated into mitochondria for aerobic oxidation, which produces a quantity of energy for cell metabolism. Nevertheless, PKM2 exists as an activated tetramer or a dimer with low activity in cancer cells. The dimer shifts cellular glycometabolism towards anaerobic oxidation to provide necessary energy, metabolic intermediate products and redox force for tumor cells and embryonic cells that are proliferating rapidly [11, 12].

Warburg effect, put forward by Germans Otto Heinrich Warburg in 1956, suggests that tumor cells tend to take anaerobic oxidation efficiently even in an aerobic condition, instead of aerobic oxidation [13, 14]. In detail, aerobic oxidation relies on tricarboxylic acid cycle (TCA) in mitochondria after the production of pyruvate in cytoplasm and ultimately get 38 molecules of ATP per glucose molecule [15]. For the normal tissues in human body, glucose predominantly participates in aerobic oxidation. Only in relatively hypoxia conditions (such as exercise), skeletal muscle cells would take anaerobic oxidation to decompose glucose [16]. However, anaerobic oxidation takes glycolysis in cytoplasm and finally gets 2 molecules of ATP per glucose molecule. For tumor cells and rapidly proliferating cells, glucose would be decomposed though aerobic glycolysis catalyzed by PKM2. In brief, PKM2 plays an important role in the glycometabolism of malignant tumors and PKM2-mediated Warburg effect can provide sufficient energy and a large amount of metabolic intermediate products for the rapid proliferation of tumor cells [17].

Recently, it is reported in numerous studies that PKM2 is instrumental in cancer occurrence, tumor proliferation and targeted therapy. Based on current research, we aim to discuss the carcinogenic effect of PKM2 with an insight into its potential to be adopted as available target for oncotherapy.

### PKM2 and Warburg effect

**Figure 1:**
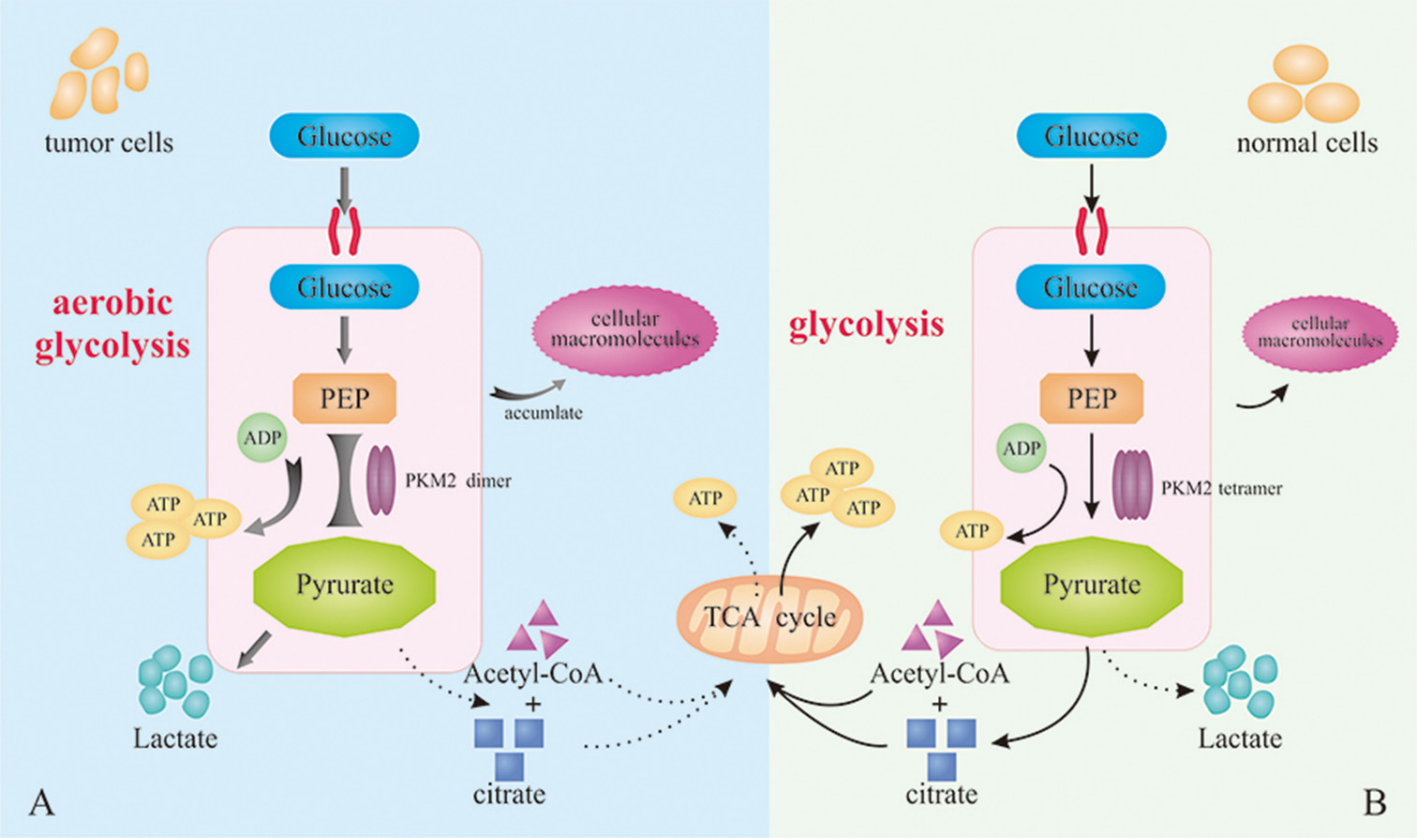
Warburg effects **(A)** PKM2 dimer mediates aerobic glycolysis to produce more lactic acid in tumor cells. **(B)** PKM2 tetramer in normal cells catalyzes the production of ATP and pyruvate for the subsequent TCA.

Both PKM1 tetramer in normal cells and PKM2 tetramer in tumor cells catalyze the transfer of phosphate from PEP to ADP, leading to the generation of ATP and pyruvate [18]. The product would participate in TCA for the production of acetyl coenzyme A and citrate. Similarly, the PKM2 dimer in tumor cells with low-activity can also catalyze the transition of PEP to ADP [19]. But compared with PKM1 tetramer and PKM2 tetramer, PKM2 dimer with significantly lower catalytic activity leads to the restriction of metabolic pathway [9]. Consequently, the accumulation of intermediate products in glycolysis provides abundant cellular macromolecules for other biosynthesis required by the rapid proliferation of tumor cells. In addition, the relative reduction of pyruvate synthesize would mediate an increase of lactic acid production [20] and remodel the tumor microenvironment, which serves as the fuel of tumor cell metabolism, promotes angiogenesis and induces immunosuppression [21, 22]. In summary, even with sufficient oxygen, PKM2 promotes the synthesis of lactic acid in most tumors through aerobic glycolysis, instead of through aerobic oxidation in mitochondria, providing energy and substantial intermediate metabolites for the proliferation of tumor cells. The biological process mentioned above is widely acknowledged as Warburg effect (Figure 1).

### The role of PKM2 in tumorigenesis, cell proliferation and glycometabolism

**Figure 2:**
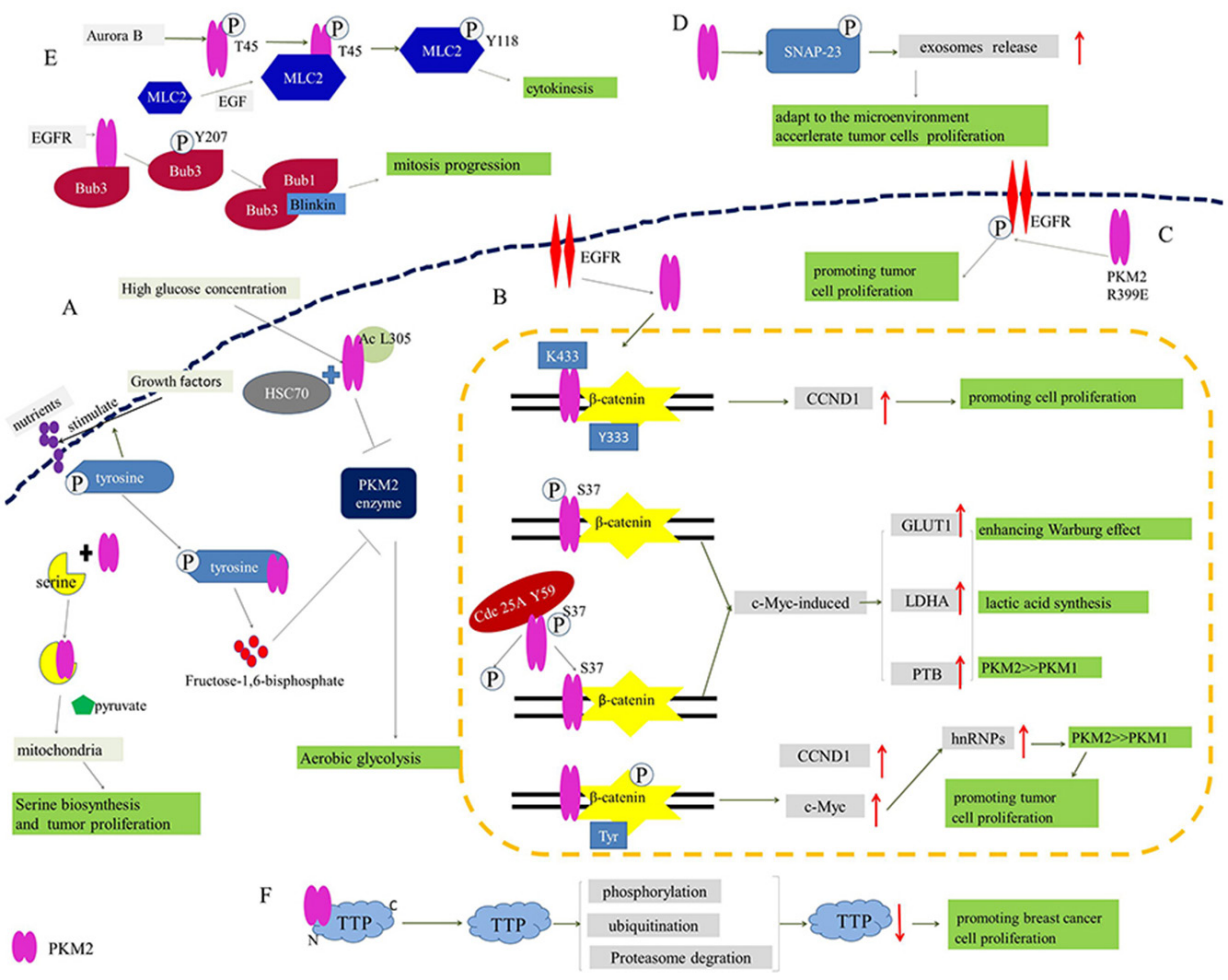
The participation of PKM2 in proliferation and glycometabolism of tumor cells **(A)** Phosphotyrosine-binding form of PKM2 relives from the inhibition of PKM2 enzyme to promote the aerobic glycolysis. **(B)** The binding of PKM2 and β-catenin regulates the expression of CCND1, GLUT1, LDHA, PTB and c-Myc to enhance Warburg effect and promote tumor cell proliferation. **(C)** R399E mutant PKM2 greatly induces the phosphorylation of EGFR to promote tumor cell proliferation. **(D)** PKM2 promotes exosomes release by the phosphorylation of SNAP-23 for the adaptation to the microenvironment and acceleration of tumor cells proliferation. **(E)** PKM2 phosphorylates MLC2 for tumor cell cytokinesis and proliferation, and EGFR activates PKM2 to phosphorylate Bub3 for mitosis. **(F)** PKM2 promotes the phosphorylation, ubiquitination and proteasome degradation of TTP for suppressing TTP protein levels to promote tumor cell proliferation.

Growth factors stimulate the cellular uptake of nutrients for biosynthesis and metabolism, which is promoted by phosphorylation of signaling proteins on the tyrosine residues [23]. Additionally, the binding of phosphotyrosine peptides and PKM2 leads to the release of fructose-1,6-bisphosphate. Fructose-1,6-bisphosphate is an allosteric activator and it would cause the inhibition of PKM2 enzymatic activity, which diverts glucose metabolites from energy production to anabolic processes [24]. In brief, the phosphotyrosine-binding form of pyruvate kinase is conducive to rapid growth of tumor cells [25]. Apart from the binding with phosphotyrosine peptides, PKM2 could be activated by serine though direct binding of them. Accompanied with the serine consumption, the activity of PKM2 decreases, which induces cells into a fuel-efficient mode. As a result, more pyruvate (produced by glycolysis) is diverted to the mitochondria and more glucose-derived carbon is applied in serine biosynthesis, ultimately maintaining tumor cell proliferation [26]. On the other hand, it is confirmed that PKM2 is acetylated at lysine 305, which is stimulated by high glucose concentration. The acetylation decreases PKM2 enzymatic activity and enhances the interaction between PKM2 and HSC70, a chaperone for chaperone-mediated autophagy (CMA), promoting its lysosomal-dependent degradation via CMA [27, 28]. Thereby, PKM2 acetylation could induce the accumulation of intermediate products of glycolysis in tumor cells, thus promoting cell proliferation and tumor growth (Figure 2A).

In accordance with Warburg effect, compared with normal cells, tumor cells give priority to anaerobic oxidation even in aerobic condition and more lactic acid is produced, which is initially mediated by PKM2 dimer in tumor cells [29]. Besides, induced by the activation of epidermal growth factor receptor (EGFR), PKM2 can be translocate into nucleus where K433 of PKM2 binds to c-Src-phosphorylated Y333 of β-catenin. Subsequently, the binding contributes to the recruitment of these two proteins by cyclin D1 (CCND1) promoter, up-regulating the expression of CCND1. PKM2-dependent β-catenin transactivation and CCND1 expression are instrumental in EGFR-promoted tumor cell proliferation and brain tumor development [30, 31]. Furthermore, the nuclear translocation of PKM2 could also be induced by EGFR-activated ERK2, and PKM2 is phosphorylated at S37 without the phosphorylation of PKM1, which recruits PIN1 for cis–trans isomerization of PKM2 and promotes the binding to importin α5 and nuclear translocation of PKM2. After the translocation of PKM2, nuclear PKM2 induces expression of c-Myc though the activation of β-catenin. Thereby the expression of three nuclear PKM2-dependent glycolytic enzymes was upregulated, including glucose transporters 1 (GLUT1), lactate dehydrogenase A (LDHA) and PTB-dependent PKM2. This will facilitate the Warburg effect and tumorigenesis ultimately [32]. Moreover, EGFR activation results in c-Src-mediated Cdc25A phosphorylation at Y59. On the one hand, the phosphorylated Cdc25A interacts with nuclear PKM2 though the dephosphorylation of PKM2 at S37. On the other hand, it promotes PKM2-dependent β-catenin transactivation and upregulated expression of the glycolytic genes (GLUT1, PKM2 and LDHA) and Cdc25A. Consequently, Cdc25A-mediated PKM2 dephosphorylation would promote the Warburg effect, cell proliferation and brain tumorigenesis [33]. In addition, PKM2 is associated with p-Tyr-β-catenin to increase transcription of c-Myc and CCND1, which could stimulate cell growth. c-Myc promotes the expression of hnRNPs, leading to alternative splicing of PK to generate PKM2 isoform or PKM1 isoform. Importantly, hnRNPs favors alternative splicing of PK to generate PKM2 isoform in tumor cells [34], indicating an involvement in enhancing Warburg effect and tumor cells proliferation (Figure 2B).

Accumulating evidences indicate that dimeric PKM2 is released from tumor cells into the circulation system of tumor patients [35]. The level of EGFR phosphorylation would significantly increase on the exposure of cells to the recombinant PKM2 protein, and the secretion of PKM2 would induce EGFR phosphorylation and activate the EGFR downstream signaling in triple-negative breast cancer cells [36]. On the contrary, PKM2 knockdown decreases EGFR phosphorylation. Interestingly, compared with wild-type PKM2, expression of R399E mutant PKM2 could more greatly enhance EGFR phosphorylation, cellular transformation and cell proliferation [37], which unveils a new effect of extracellular PKM2 on promoting cancer cell proliferation through EGFR activation (Figure 2C).

To communicate with the microenvironment, tumor cells would actively release a lot of extracellular vehicles (EVs) including microvesicles (MVs), exosomes, apoptotic bodies or microparticles that are involved in the remodeling of the tumor-stromal environment and promoting malignancy. Namely, they play a significant role in promoting tumor rapid proliferation [38, 39]. Meanwhile, it is reported that PKM2, serving as a protein kinase, promotes exosomes release by the phosphorylation of synaptosome-associated protein 23 (SNAP-23), which in turn enables the formation of the SNARE complex [40]. In short, PKM2 could promote tumor cell exosomes release for the adaptation to the microenvironment and acceleration of tumor cells proliferation (Figure 2D).

What's more, it has been found that PKM2 with high expression in embryonic development and tumor progression is phosphorylated at T45 by Aurora B, which leads to PKM2 localization and its interaction with myosin light chain 2 (MLC2) in the contractile ring region of mitotic cells during cytokinesis. In detail, PKM2 phosphorylates MLC2 at Y118, which is notably enhanced by EGF stimulation or EGFRvIII, K-Ras G12V and B-Raf V600E mutant expression. And this is also crux for cytokinesis, cell proliferation and tumor development. In short, PKM2 is involved in cytokinesis in malignant tumors, ultimately forming a new “molecular label” pattern to regulate cell proliferation [41]. In contrast, without the regulation of mitotic checkpoints by PKM2, tumor cells will not be split successfully. Additionally, the depletion of PKM2 can lead to uneven distribution of DNA into two new cells, triggering the death or apoptosis of programmed cells after cells division. During mitosis, PKM2, activated by EGFR, binds to the spindle checkpoint protein Bub3 then phosphorylates it at Y207. And the phosphorylation of Bub3 is required for recruitment of Bub3-Bub1 complex to kinetochores where it interacts with Blinkin [42]. And this is essential for correct kinetochore-microtubule attachment, mitotic/spindle-assembly checkpoint, accurate chromosome segregation, cell survival and proliferation, and active EGFR-induced tumorigenesis. Beyond that, the phosphorylation of Bub3 also correlates with histone H3-S10 phosphorylation in human glioblastoma mentioned above. Taken together, PKM2 plays a vital role in tumor cell division and proliferation (Figure 2E) [43].

Tandem zinc finger protein tristetraprolin (TTP), an AU-rich element (ARE)-binding protein, belongs to TIS11/TTP gene family [44]. In addition to its function in immune response, TTP is also involved in tumorigenesis, cell differentiation and apoptosis [45, 46]. In fact, TTP is well- known as a tumor inhibitor, while PKM2 has a property of tumor support. It is reported in the previous study that some kinases, including protein kinase B (PKB)/AKT, p38 mitogen-activated protein kinase (MAPK), MK2 and extracellular signal-regulated kinase 1 (ERK1), can phosphorylate TTP. Therein, P38 MAPK/MK2 pathway crucially regulate TTP [47]. Intriguingly, PKM2 could promote the phosphorylation, ubiquitination and proteasome degradation of TTP though its combination with N-terminus of TTP, ultimately suppressing TTP protein levels (Figure 2F). In contrast, the inhibition of p38 MAPK by siRNA or inhibitor SB203580 could prevent the degradation of TTP and weaken the effect of PKM2 simultaneously, which indicates that the p38 MAPK/MK2 pathway might be involved in PKM2-mediated TTP degradation. Moreover, the manipulation of PKM2 protein level is closely tied with the degradation of TTP mRNA [48]. Given the above, it can be concluded that the association between PKM2 and TTP is pivotal during cell proliferation.

### The role of PKM2 in angiogenesis

**Figure 3:**
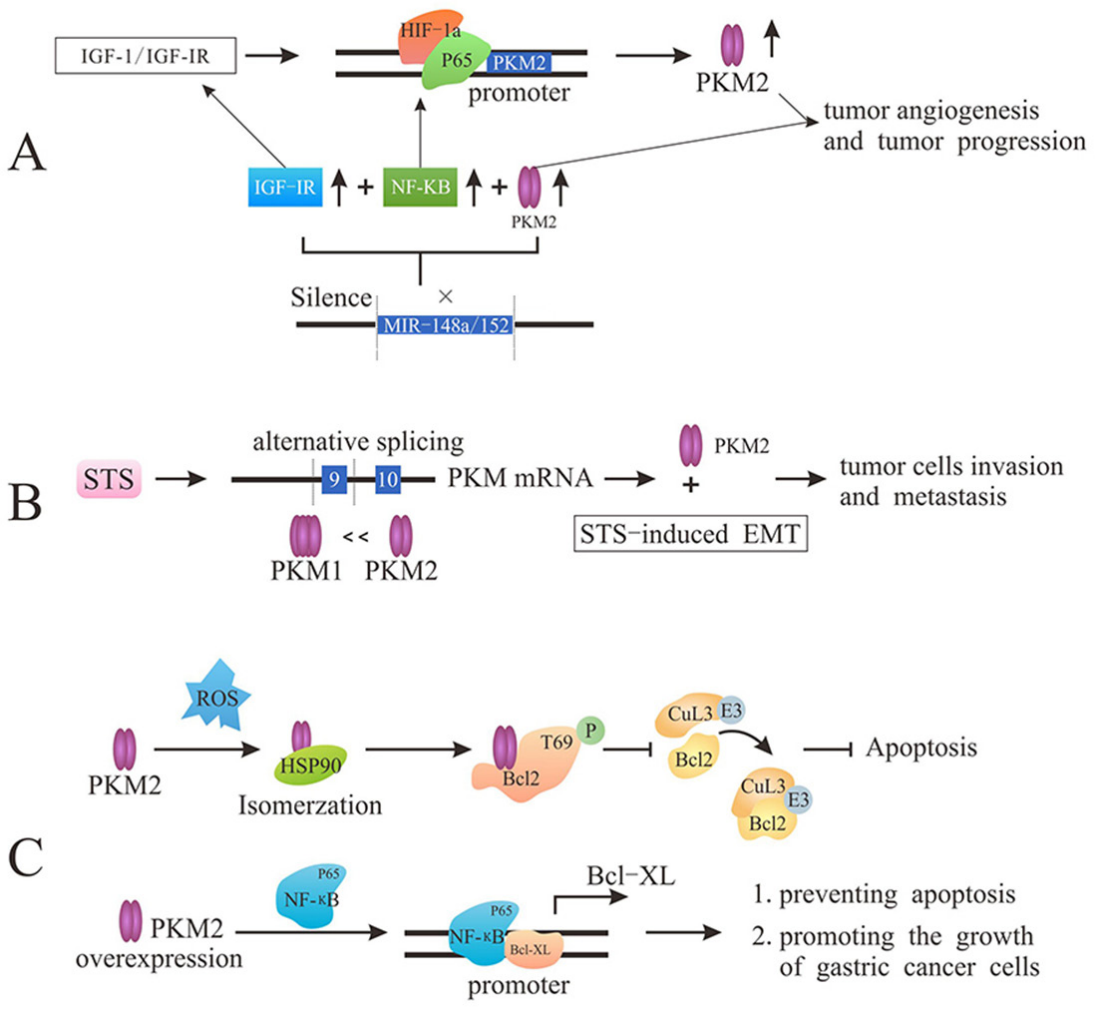
PKM2 promotes tumor angiogenesis and metastasis and prevents apoptosis **(A)** PKM2 regulates tumor angiogenesis though either the enhancement of endothelial cell proliferation, migration, and cell-ECM adhesion or PKM2/NF-κB/miR-148a/152 feedback loop. **(B)** STS induces PKM2 via PKM genes alternative splicing to stimulate cancer cell invasion and migration. **(C)** Under oxidative stress, the interaction with HSP90 induces PKM2 conformation and translocation into mitochondria, and PKM2 phosphorylates Bcl2 prevent the binding of Cul3-based E3 ligase to Bcl2, leading to the degradation of Bcl2 and the inhibition of apoptosis. Overexpression of PKM2 promotes the binding of p65 to Bcl-xL promoter to up-regulate the expression of Bcl-xL protein, which ultimately prevents apoptosis and promotes the growth of gastric cancer cells.

Tumor angiogenesis is an extremely complex process, including vascular endothelial matrix degradation, endothelial cell migration and endothelial cell proliferation [49]. The newborn tumor vasculature provides nourishment for the constantly infiltration and growth of tumor cells. Importantly, PKM2 (the dimer, not the tetramer) in the blood could promote tumor angiogenesis by enhancing endothelial cell proliferation, migration, and cell-ECM adhesion, eventually facilitating tumor growth [17, 50]. Distinct from the mechanism mentioned above, it is also reported that the disruption of PKM2/NF-κB/miR-148a/152 feedback loop can regulate cancer cell growth and angiogenesis, particularly in triple-negative breast cancer (TNBC) phenotype. The expression of PKM2 is regulated by IGF-1/IGF-IR via enhancing the binding of HIF-1α-p65 complex with PKM2 promoter and also up-regulated by miR-148a/152 suppression [51]. And in some cancer cells, the silencing of miR-148a/152 contributes to the overexpression of PKM2, NF-κB and IGF-IR that play critical role in the tumor development and metabolism [52]. Together, PKM2 plays a momentous role in tumor cells proliferation and tumorigenesis by participating in PKM2/NF-κB/miR-148a/152-modulated tumor angiogenesis and tumor progression (Figure 3A).

### The role of PKM2 in metastasis

In addition to infinite proliferation, malignant tumor cells are also characterized by infiltration and metastasis [53]. It is demonstrated that human steroid sulfatase (STS) could enhance PKM2 expression. Overexpression of STS induces PKM2 and suppresses PKM1 in both mRNA and protein level via regulating PKM genes alternative splicing. PKM2 overexpression could stimulate cancer cell invasion and migration, but PKM2 knockdown would hamper the STS-induced cell invasion and migration. Moreover, PKM2 is highly associated with STS-induced epithelial mesenchymal transition (EMT), which is related to migration and invasion of cancer cells [54, 55]. In a word, PKM2 can highly promote the STS-mediated cancer cell invasion and metastasis (Figure 3B).

### The role of PKM2 in the apoptosis

Tumor cells exhibit more oxygen species (ROS) than normal cells due to oncogenic stimulation and mitochondrial malfunction, inducing tumor cells apoptosis. In the condition of oxidative stress, tumor cells become well-adapted to such stress through activating ROS-scavenging systems and ultimately inhibit apoptosis [56]. After interacting with chaperone protein HSP90 in cytoplasm, PKM2 would change its conformation and translocate into mitochondria where PKM2 interacts with Bcl2 (encoded by Bcl-2 proto-oncogenes) and phosphorylates Bcl2 at T69. The phosphorylation of Bcl2 could prevent the binding of Cul3-based E3 ligase to Bcl2 and subsequent degradation of Bcl2, ultimately inhibiting the apoptosis induced by oxidative stress [57]. In other words, PKM2-mediated Bcl2 phosphorylation plays a stimulative role in the occurrence and development of glioblastoma though enhancing apoptosis resistance of tumor cells and promoting tumorigenesis. Furthermore, the overexpression of PKM2 enhances the stability of NF-κB subunit p65, promoting the binding of NF-κB subunit p65 to Bcl-xL promoter [58]. Thereby, PKM2 could up-regulate the expression of Bcl-xL protein (anti-apoptotic member of Bcl-2 protein family) at the transcriptional level, thus preventing apoptosis and promoting the growth of gastric cancer cells (Figure 3C).

### The role of PKM2-related genes in tumor cells

**Figure 4:**
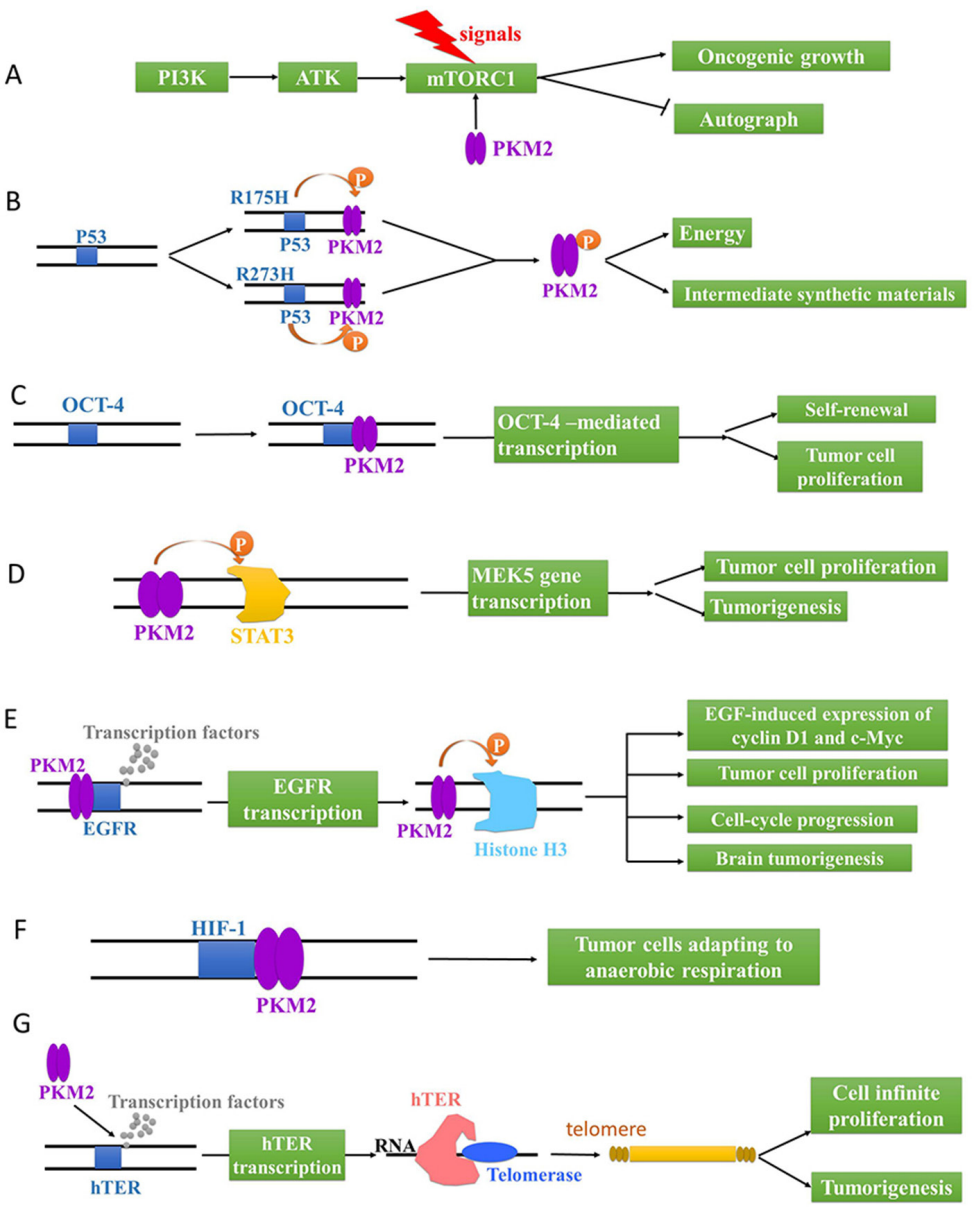
The genetic regulation via PKM2 in tumor cells **(A)** PKM2 activates mTORC1 to accelerate oncogenic growth and inhibit autophagy. **(B)** Mutant R175H and R273H of P53 phosphorylates PKM2 to provide energy and intermediate synthetic materials for tumor cells. PKM2 activates OCT-4 **(C)** and MEK5 **(D)** gene transcription to promote the self-renewal and proliferation of tumor cells. **(E)** PKM2 activates EGFR gene transcription to phosphorylate Histone H3 for EGF-induced expression of cyclin D1 and c-Myc, tumor cell proliferation, cell-cycle progression, and brain tumorigenesis. **(F)** PKM2 activates HIF-1, promoting tumor cells to adapt to anaerobic respiration. **(G)** PKM2 up-regulates hTERT gene transcription to catalyze the synthesis of telomere repeat sequence at the end of chromosome for cell infinite proliferation and tumorigenesis.

The mutations of proto-oncogenes and tumor suppressor genes that controll metabolic pathways may lead to abnormal metabolism and rapid proliferation of tumors. It has been reported that the PI3K/AKT/mTOR pathway plays an incomparable effect in regulating cell proliferation and energy metabolism. The overexpression of PI3K and ATK, two proto-oncogenes, would trigger tumors, whereas the mammalian target of rapamycin complex 1 (mTORC1), responsible for integrating environmental and intracellular signaling, could regulate cell growth [59]. In tumor cells, mTORC1, activated by hormone and nutritional signaling, controls a series of macromolecule biosynthetic processes, such as mRNA transcription, protein translation and lipid biosynthesis. Intriguingly, PKM2 phosphorylates AKT1 substrate 1 (AKT1S1, mTORC1 inhibitor) at S202 and S203, which results in hormonal and nutritional signaling-independent activation of mTORC1 [60]. Consequently, the activated mTORC1 would lead to the acceleration of oncogenic growth and autophagy inhibition in tumor cells (Figure 4A).

On the other hand, TP53 gene, is classified as a tumor suppressor gene, frequently mutates into mutation P53 in aggressive malignant tumor cells. Apart from wild-type P53, mutant P53 isoforms also display notably carcinogenic effect and are referred to as GOFs (gain of functions), which culminates in chemoresistance to tumor therapies, genomic instability and aberrant deregulation of cell cycle progression [61]. Particularly, mutant R175H and R273H of P53 could phosphorylate PKM2 at T105 through mTOR signaling pathway. In turn, the activation of mTOR/PKM2 pathway would maintain the oncogenic activity of mutant P53 [62]. Namely, the interaction between mutant P53 and PKM2 would provide energy and intermediate synthetic materials for tumor cells through aerobic glycolysis (Figure 4B).

Moreover, OCT-4 gene, also known as the pluripotent factor of stem cells, plays an important role in maintaining the pluripotent state of embryonic stem cells. And the expression of OCT-4 gene was significantly enhanced in rapidly proliferating cells. Surprisingly, PKM2 (C-terminal domain, amino acids 307-531) can bind to OCT-4, enhancing OCT-4-mediated transcription, which promotes the self-renewal and proliferation of tumor cells (Figure 3C) [63]. Similarly, cell proliferation and tumorigenesis could also be accelerated by the activated MEK5 gene transcription after the phosphorylation of STAT3 705-bit tyrosine by PKM2 (Figure 4D) [64].

With the activation of EGFR via growth factors, PKM2 proteins are translocated into the nucleus where they bind to the promoter region of EGFR gene, and transcription factors can also be recruited by the promoter region to initiate genetic transcription. As a result, PKM2 directly binds to histone H3 and phosphorylates it at T11 upon EGFR activation, which is also indispensable for histone acetyl enzyme 3 (HDAC3) to dissociate from the CCND1 and Myc promoter regions [65]. HDAC3 subsequently acetylates histone H3 at K9. Critically, PKM2-phosphorylated histone H3 T11 is related to nuclear PKM2 expression levels, glioma malignancy grades, and prognosis. Meanwhile, PKM2-mediated histone H3 modifications contribute to EGF-induced expression of CCND1 and c-Myc, tumor cell proliferation, cell-cycle progression, and brain tumorigenesis [65]. Taken together, PKM2 plays an irreplaceable role in its non-metabolic functions of histone modification and epigenetic regulation of gene expression and tumorigenesis (Figure 4E).

Besides, PKM2 is involved in a positive feedback loop where PKM2, transcriptionally activated by hypoxia-inducible factor 1 (HIF-1), could also promotes the transactivation of HIF-1 through the direct interaction with HIF-1α subunit. And the products encoded by HIF genes were involved in a variety of physiological functions including angiogenesis, nucleoside and amino acid synthesis, energy metabolism and cell proliferation in order to maintain tissue and cell homeostasis under hypoxia conditions [66, 67]. In a word, the PKM2-mediated HIF-1 activation would promote tumor cells to adapt to anaerobic respiration, providing continuous energy and intermediate metabolites for tumor cell proliferation (Figure 4F).

In addition, accompanied by the reverse transcription catalysis of human telomerase reverse transcriptase (hTERT), the telomerase would make use of RNA as a template to synthesize the telomere repeat sequence to the end of chromosome [68]. And it also prolongs or stabilizes the telomere with shortened cell division, playing an important role in the cell infinite proliferation and tumorigenesis. Crucially, hTERT is activated in most malignant tumors and makes tumor cells immortalized (Figure 4G) [69, 70]. PKM2 positively regulates the transcriptional activity of hTERT by enhancing the association of transcription factor of hTERT (SPl) with the hTERT promoter [1, 71, 72]. Therefore, it is speculated that PKM2 may modulate tumor cell growth and tumorigenesis via the interaction with hTERT.

### The role of PKM2 in oncotherapy

Most malignant tumors are the lethal diseases that endanger the health and life quality of patients. And the development of anti-tumor drugs is still a serious problem that is urgent to be solved. With the rapid development of oncobiology and related disciplines, the direction of tumor treatment has been subvert from traditional cytotoxic drugs to targeted therapy of tumorigenesis mechanism. On the basis of accurately distinguishing the difference between normal and tumor cells, the target-specific antineoplastic drugs have advantages of high selectivity and low toxicity [73]. As mentioned in present review, PKM2 is involved in the process of tumorigenesis and tumor progression, including angiogenesis, cell cycle regulation, tumor microenvironment and metabolic abnormality [22, 25, 32, 39, 50]. Besides, it is also reported that PKM2 and HSPA5 may play an important role in the progression of endometrial carcinoma (EC) as potential biomarkers in the prediction of high-risk EC, thereby guiding clinical therapy [74]. According to the important role of PKM2 in tumor, we overviewed the application of PKM2 in oncotherapy.

#### PKM2 as a potential target in oncotherapy

It is indicated that tyrosine kinase is closely associated with tumorigenesis and tumor proliferation. Over 50% proto-oncogenes encode products with protein tyrosine kinase (PTK) activity, directly causing carcinogenesis due to the abnormal expression of proto-oncogenes. Besides, growth factors stimulate cells to uptake abundant nutrients and use them for the biosynthesis and metabolism, which is initiated by phosphorylation of signaling proteins on the tyrosine residues. Because PKM2 is a phosphotyrosine-binding protein, PKM2 plays a pivotal role for rapid growth in tumor cells, providing a novel idea for oncotherapy though regulating the expression of PKM2 [25]. On the other hand, during the rapid tumor proliferation, simple osmotic effect is insufficient to meet the demand of nutrient and metabolites required by the proliferation of tumor cells. Meanwhile, the increasing expression of vascular growth factors stimulates the activation of endothelial cell and angiogenesis, thus replenishing nutrients for the tumor proliferation [75]. It is mentioned that PKM2 could promote angiogenesis through various pathways [50, 52, 76], providing evidence for the application of PKM2 inhibitor in oncotherapy.

The stimulation of the growth factors is transmitted into cells, which constitutes a complex signal transduction system that regulates the proliferation and differentiation of the tumor cells. Among them, PI3K/AKT/mTOR and Ras-MAPK pathway are closely related to tumorigenesis and development. PKM2 interacts with these two signaling pathways with high affinity [60], indicating that PKM2 might be a new target for tumor treatment. Cell division, consisting of interkinesis and mitosis, is controlled by regulatory mechanism of cellular cycle. There is a group of cyclin-dependent kinases (CDKs) in the core of regulatory mechanism, each of which is activated by the phosphorylation of substrate at a specific time, driving the completion of the cell cycle. Surprisingly, the proliferation of tumor cells is also controlled by the same regulatory mechanism [31, 41]. The role of PKM2 on tumor cell proliferation cannot be neglected, thus providing a new strategy for tumor treatment. Furthermore, the expression of histone deacetylase (HDAC) inhibitor raised by transcription factors hampers the expression of certain normal genes, which is quite common in tumors. Thus, HDAC could be a promising target for anti-tumor drugs. Besides, PKM2 can combine with histone H3 and be adsorbed to T11-level phosphate group after EGFR activation, which promotes the proliferation of tumor cells [65, 77]. Therefore, it is suggested that PKM2 plays a similar role with HDAC as a potential therapeutic target for tumor therapy.

In addition, cancer stem cell/tumor-initiating cell (CSC/TIC), a cell subset with characteristics of stem cell, possesses obviously stronger proliferative ability compared with other cancer cells in the same tumor tissues. And it essentially participates in the occurrence, development and maintenance of tumors, and promotes carcinogenesis in animals. Crucially, the regulation of PKM2 can promote CSC/TIC differentiation and induce apoptosis [57], indicating that PKM2 could be an oncotherapy target. Moreover, mitochondria are the main energy-supplying units of normal cells. But tumor cells, even under aerobic conditions, principally acquire energy though aerobic glycolysis (Warburg effect) [20–22]. Namely, the energy supply of tumor cells is predominantly dependent on glycolysis, in which PK is the rate-limiting enzyme. Therefore, the regulation of PKM2 could be a potential target in the angle of tumor metabolism. Additionally, it is substantiated that the reactivation of hTERT is associated with tumor growth, which is an essential feature of malignant tumors [70]. Notably, PKM2 regulates hTERT transcription by influencing the combination of the upstream transcription factor SPL with the hTERT promoter, thus facilitating the proliferation of hepatocellular carcinoma cells [71, 72]. Thereby, it is hypothesized that through the regulation of PKM2 by taking some effective measures, we can control the transcription of hTERT to achieve the goal of treating tumors.

#### The inhibition of PKM2 in oncotherapy

**Figure 5:**
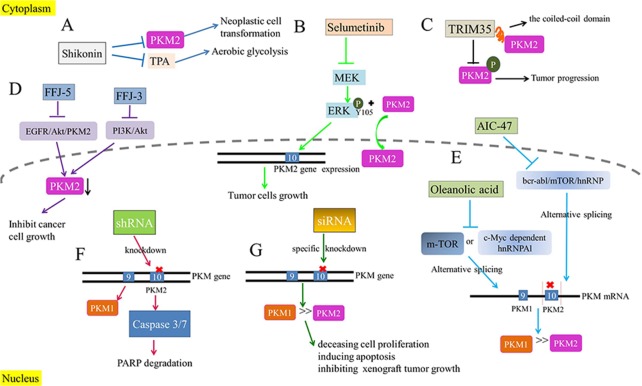
The inhibition of PKM2 as a therapeutic target **(A)** Shikonin inhibit TPA-induced neoplastic cell transformation and PKM2 to suppress aerobic glycolysis. **(B)** Selumetinib as an MEK's inhibitor decreases the expression of PKM2 and the production of lactic acid to suppress tumor cell proliferation. **(C)** TRIM35 interacts with the coiled-coil domain of PKM2 to alleviate the tumor cell proliferation. **(D)** FFJ-5 and FFJ-3 down-regulate PKM2 to inhibit cancer cell growth. **(E)** OA alternatively splices PKM transcriptional initial mRNA and AIC-47 disrupts Bcr-abl/mTOR/hnRNP signaling pathway to produce more PKM1 and abolish Warburg effect. **(F)** shRNA is used to knockdown PKM2 gene with PKM1 substitution for suppressing Warburg effect. **(G)** siRNAs is used to silence PKM2 gene for the inhibition of tumor cells.

Most of tumors are characterized by abnormal metabolism and elevated expression of PKM2, which is also known as Warburg effect. Higher expression of PKM2 in tumor cells compared with normal cells suggests that PKM2, one of the important metabolic enzymes, might serve as a significant target of tumor treatment. It is demonstrated that specific PKM2 inhibitors can inhibit the growth and survival of tumor cells [78]. Herein, Shikonin, a small-molecule active chemical, serves as a PKM2 inhibitor and has been applied as an anti-cancer drug in human cancer models [79]. In drug-sensitive and resistant tumor cell lines that primarily express PKM2, it is manifested by the determination of cellular lactate production and glucose consumption that Shikonin (alkannin) significantly suppresses the glycolytic rate [80]. Moreover, it is ascertained that Shikonin could suppress the tumor promoter 12-O-tetradecanoylphorbol 13-acetate (TPA)-induced neoplastic cell transformation and PKM2 activation in the early stage of carcinogenesis (Figure 5A) [81].

Apart from the PKM2 inhibitors above directly restraining the activity of PKM2, there are still some inhibitors indirectly weakening the effect of PKM2 though intermediate molecule. PKM2 could activate genes involved in cell proliferation and Warburg effect after the translocation into nucleus, which suggests a novel orientation for oncotherapy. It is validated that activated-MEK protein could activate ERK (attached to PKM2 though a phosphate group), promoting the translocation of PKM2 into nucleus and high expression of it. Importantly, with MEK/ERK inhibitors, the process above would be prevented, namely, EGF-induced nuclear translocation of PKM2 would be blocked. Selumetinib, MEK's inhibitor, can reduce the phosphorylation of ERK. Ultimately it would decrease the expression of PKM2 and the production of lactic acid, therefore, the tumor cell growth would be suppressed (Figure 5B) [32]. On the other side, tripartite motif-containing protein 35 (TRIM35), identified as a novel tumor suppressor in human hepatocellular carcinoma (HCC), could interact with PKM2 upon the coiled-coil domain. Interestingly, the coiled-coil domain mediates the alleviation of Warburg effect and proliferation of HCC cells. Simultaneously, TRIM35 inhibits the tumorigenicity of HCC cells by blocking PKM2 Y105 (tyrosine residue 105) phosphorylation, because PKM2 has been demonstrated to have a central role in metabolic reprogramming after it is up-regulated by phosphorylation of PKM2 Y105 during cancer progression [82]. Collectively, TRIM35 provides a new therapeutic regimen for tumors though the interaction with PKM2 (Figure 5C). Besides, researchers have found that as the structurally modified versions of mollugin, FFJ-5 and FFJ-3 can inhibit cancer cell growth through down-regulation of PKM2 [85, 86]. FFJ-5, a naphthoquinone modifier of mollugin, attenuates the expression of PKM2 and reduces the production of ATP via blocking the EGFR-Akt-PKM2 pathway, which eventually induces tumor cell apoptosis [83]. And FFJ-3 inhibits PKM2 expression via suppressing PI3K/Akt signaling pathway and activates the mitochondrial apoptosis signaling pathway in human cancer cells [84] (Figure 5D). Therefore, we conclude a potential therapeutic role for FFJ-5 and FFJ-3 in the treatment of human cancer.

In addition to the direct and indirect effects of certain inhibitors on PKM2, the regulation of PKM genetic expression could also generate the similar effect with PKM2 inhibitors. It is found that oleanolic acid (OA) alternatively splices initial PKM transcriptional mRNA to produce more PKM1 instead of PKM2 by blocking mTOR signaling pathways or c-Myc-dependent hnRNPAl, which abrogates Warburg effect in tumor cells [85]. In other words, OA can suppress aerobic glycolysis, indicating that OA could be developed as a novel anticancer agent for oncotherapy. Similarly, it is also reported that AIC-47, a fatty-acid derivative, can lead to the disruption of Bcr-abl/mTOR/hnRNP signaling pathway and the expression switching from PKM2 to PKM1 [85] (Figure 5E). Besides, researchers use short hairpin RNA (shRNA) to knockdown PKM2 gene in human cancer cells with PKM1 substitution. As a result, it is observed that production of lactic acid decreases and oxygen consumption increases in tumor tissues of xenograft mice, indicating that Warburg effect is suppressed [11]. Similarly, the knockdown of PKM2 gene promotes the activity of caspase-3/7 and induces the degradation of poly ADP-ribose polymerase (PARP), a DNA repair enzyme [87]. Thereby, it is confirmed that the inhibition of PKM2 has a tremendous potential as a therapy for glioblastoma (Figure 5F). Furthermore, siRNA with inherent nucleotide-level specificity can be utilized in oncotherapy, because it can target isoform-specific exons in genes that exhibit differential splicing patterns in various cell types. Due to a target mismatch between the M2 and M1 isoform, siRNAs confers specific knockdown of the former, culminating in the decease of cell proliferation, induction of apoptosis and inhibition of xenograft tumor growth [88]. Taken together, this technique may provide an effective way for human to treat cancers and reduce side effects at the same time (Figure 5G).

#### PKM2 activators in oncotherapy

**Figure 6:**
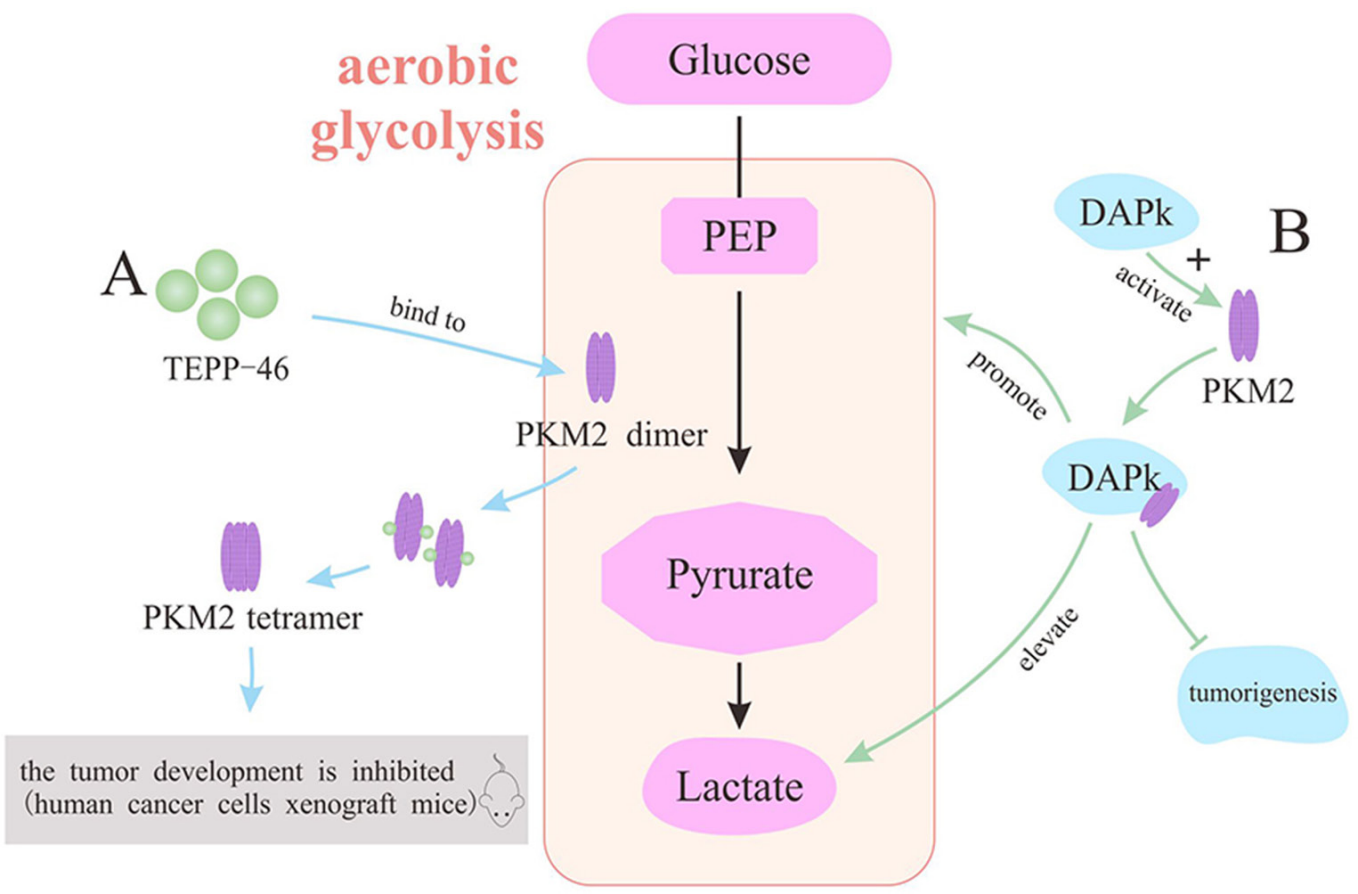
The activators of PKM2 for the treatment of tumors **(A)** TEPP-46 activates PKM2 to impair tumorigenesis. **(B)** DAPk activates PKM2 to restrain cell growth.

It is still controversial to use PKM2 as an efficient target in the treatment of malignant tumors, although PKM2 inhibitors have made great progress in providing a new strategy for curing cancers. On the contrary, it is unexpectedly reported that PKM2 is associated with the inhibition of tumor growth in a mouse breast cancer model, suggesting that pyruvate kinase with high activity might suppress tumor growth by transferring the carbohydrate metabolic intermediates from biosynthesis to energy production [89]. Based on the precious study, synthetic PKM2 activators can increase the activity of PKM2 in cells, and it is comparable to the activity of PKM1 by switching PKM2 from dimer to tetramer. Specifically, PKM2 activators bind to a pocket of PKM2 subunit interface, thereby enhance the association between PKM2's subunits to form stable tetramers [16]. TEPP-46, a member of the thieno[3,2b]pyrrole[3,2-d] pyridazinones class, is a small molecular activator of PKM2 demonstrated by a controlled experiment in mice. The development of tumors in human cancer cells xenograft mice is hindered both in the group of solely expressing PKM1 and the group of continuous dosing with TEPP-46 [58, 90]. The positive result indicates that PKM2 with increasing activity might impair tumorigenesis (Figure 6A). Moreover, there are some activators that promote the expression of PKM2 via different mechanisms and ultimately slack the proliferation of tumors. Death-associated protein kinase (DAPk), a multi-domain serine/threonine kinase, could regulate death mechanisms of tumor cells, manifesting that DAPk can function as tumor suppressor [91]. It is certified that full-length DAPk directly binds to and functionally activates PKM2, which contributes to the regulation of tumor cell metabolism [92]. Notably, with a truncated DAPk lacking kinase domain (KD), the PKM2's endogenous activity is also elevated, suggesting that the activation of PKM2 by DAPk is independent of the kinase activity. Importantly, DAPk-transfected cells display changes in glycolytic activity, including the elevation of lactate production and the mild reduction of cell proliferation, whereas glucose uptake remains unaltered [93]. Altogether, it is identified that DAPk, as a novel metabolic regulator and tumor suppressor, directly interacts with key glycolytic enzyme in order to restrain cell growth. Hopefully, it provides a new insight into mechanism underlying potential therapeutic target of tumors (Figure 6B).

#### The regulatory effects of miRNAs on PKM2 in oncotherapy

MicroRNAs (MiRNAs), noncoding RNAs with specific down-regulatory role in target gene expression, are found in all eukaryotic cells conserved across the species in 1993 [94]. In recent decade, the regulation of miRNAs is extensively studied for their role in biological processes and progression of many human diseases, especially in cancer. Importantly, it is suggested that miRNAs could restrain PKM2 directly or indirectly.

On the one hand, the alternative splicing of PK is controlled by heterogeneous nuclear ribonucleoprotein (hnRNP) family members hnRNPA1, hnRNPA2, and PTB1 [85], inducing the switching of PKM isoform from PKM2 to PKM1 [95]. Consequently, the up-regulation of PTB1 are found to function as a tumor suppressor in colorectal and pancreatic cancer [96–98]. Intriguingly, the ectopic expression of miR-124 could induce apoptosis and autophagy of colon cancer cells by targeting PTB1 [97]. Besides, miR-137, miR-340, miR-1, and miR-133b have essential roles in down-expression of cancer-dominant PKM2 by the regulation of PTB1 in colorectal cancer [97, 99]. Moreover, miR-145 directly binds to 3'UTR of KLF4 and negatively regulates Warburg effect by silencing KLF4 and PTB1 in bladder cancer cells, resulting in significant cell growth inhibition [100]. Obviously, it is still controversial in the regulatory mechanism of PKM2 through PTB1-associated miRNAs, requiring further research for cancer therapy.

On the other hand, some studies confirm that miR-let-7a could inhibit cell proliferation, metastasis, and invasion by down-regulating PKM2 in gastric, glioma, and cervical cancer [101–103]. Namely, PKM2 is highly expressed in gastric and cervical cancer tissues and possesses a strongly inverse correlation with the expression of miR-let-7a which is a tumor suppressors [101, 102]. Unfortunately, the specific regulatory mechanism of miR-let-7a to PKM2 is still unclear. In addition, c-Myc promotes up-regulation of hnRNPA1 expression which would bind to PKM pre-mRNA and induce PKM2 expression. MiR-let-7a functionally targets c-Myc, down-regulates the pathway above, and eventually inhibits cell proliferation and aerobic glycolysis in glioma cells [103]. Notably, PKM2 is also a direct target of miR-122 and the overexpression of miR-122 reduces both the mRNA and protein levels of PKM2, suggesting therapeutic intervention of miR-122 in hepatocellular carcinoma [104]. What's more, abnormal expression of miRNA-326 in glioma cells could induce apoptosis and reduce metabolic activity by targeting PKM2 [87].

Taken together, miRNAs may become a new biological marker of tumor diagnosis, a molecular drug targets, and a molecular simulation for new drug research and development. However, the efficient delivery of miRNAs to target tissues is apparently a major challenge in the transition of miRNA therapy to clinical application.

## CONCLUSION

It is well-known that PKM2, a pyruvate kinase, participates in glycolysis and provides energy and intermediate products for other biosynthesis required by the rapid proliferation of tumor cells. Namely, PKM2 plays an effective role on glucose utilization, glucose uptake, lactic acid production in the glucose metabolism of tumor cells and a significant role in the transformation of many malignant cancers, including facilitating Warburg effect, preventing apoptosis, enhancing proliferation, and promoting cancer angiogenesis. In addition to regulating glycometabolism, PKM2 is involved in the expression of numerous PKM2-related gene and key components of tumor cell development, thus directly or indirectly regulating proliferation, apoptosis and metastasis of tumor cells. Based on its facilitation on tumor progression, PKM2 is predicted to be a clinical prognostic indicator for many malignant cancers such as hepatocellular carcinoma, gastric cancer, tongue cancer, gallbladder cancer and esophageal squamous cell carcinoma.

Due to the pivotal role of PKM2 in glycometabolism and various cellular processes of tumor cells, targeting PKM2 might be an ideal therapeutic strategy for malignant tumors that are characterized by infinite proliferation, metastasis and chemoresistance. Given this, artificially inhibiting the activity of PKM2 may drive the restrain of tumor cells growth. Because of high expression and specificity in tumor cells, PKM2 is preferentially considered to be a potential biomarker for tumor diagnosis and monitoring.
